# Core influencing factors of group blood donation in Beijing

**DOI:** 10.3389/fpubh.2025.1650567

**Published:** 2026-01-23

**Authors:** Rui Liu

**Affiliations:** Beijing Red Cross Blood Center, Beijing, China

**Keywords:** group blood donation coordinators, group blood donation incentives, group blood donors, group entity leadership, social environment

## Abstract

**Introduction:**

Group blood donation is a significant component of the urban blood supply. The imperative to enhance the management and utilization of this workforce, to increase the organizational mobilization capabilities of various blood donation entities, and to identify the pivotal factors influencing the successful mobilization of group blood donation efforts, thereby augmenting the total volume of group blood donations across the city, represents a critical research topic.

**Methods:**

This article adopts the organizational mobilization model of group blood donation as the entry point. A comprehensive investigation was conducted on the role of leadership prioritization within entities, the competencies of individuals responsible for managing group blood donations, the level of incentives provided to donating employees, and the engagement level of blood donors. This study retrospectively evaluates and ranks the importance of these four indicators across 100 blood donation entities in Beijing.

**Results:**

The study revealed that the importance of leadership prioritization in blood donation (79%) emerged as the primary factor influencing successful group blood donation, followed by the level of incentives provided to blood-donating employees, the engagement level of blood donors, and the competence of the individuals responsible for organizing group blood donations.

**Conclusion:**

Among the four core factors influencing the mobilization of group blood donations, prioritization by entity leadership toward group blood donation activities is identified as the most crucial. The study also found that the broader social environment significantly impacts group blood donation. A favorable social environment facilitates group blood donation activities, whereas adverse conditions, such as severe infectious disease outbreaks or negative public perception toward voluntary blood donation, can detrimentally affect these activities.

## 1 Background and problem statement

Group blood donation represents a collective approach to blood donation. The Beijing Municipal Health Commission mandates that district health commissions implement blood donation campaigns and recruitment efforts in accordance with the *Beijing Blood Donation Regulations* (1). Participants in group blood donation encompass a wide variety of institutions including government agencies, local community offices, healthcare facilities, corporate entities, public institutions, universities, municipal state-owned assets commissions, community organizations, and military units.

At the start of each year, these group entities receive a directive from the Beijing Blood Donation Office entitled “*Notification on Ensuring the Annual Voluntary Blood Donation Work*”. District health commissions are charged with the responsibility of mobilizing and recruiting group blood donations within their respective jurisdictions. The leadership of these group entities delegates the task of organizing blood donation efforts to designated coordinators within their organizations. These coordinators are responsible for mobilizing and recruiting participants from their respective institutions. Although there is considerable variability in the nature, type, and industry of the group entities, the organizational model for mobilizing donations remains largely consistent.

All blood donation entities throughout the city receive the same mobilization and recruitment document, yet the outcomes in terms of blood donation volumes differ markedly. Identifying the factors that influence the final number of blood donors is critical for understanding the dynamics of recruitment to collection in the blood donation process. This research could potentially enhance the success rate of mobilization for group blood donations and increase blood collection.

The types of entities involved in group blood donations vary, and entities of the same type may exhibit significant differences in their operational contexts. The broader societal context also changes frequently, and factors such as pandemics and public sentiment can negatively impact group blood donation efforts. Nevertheless, some blood donation entities successfully meet their donation targets despite these adverse conditions. The researcher posits that certain core factors underpin the success of group blood donations. Identifying these key factors is vital for ensuring the smooth completion of group blood donation activities.

## 2 Literature review

### 2.1 The emphasis on group blood donation by leadership

Within an organization, leadership is pivotal in decision-making and guiding practices, with employees generally adhering to the directives and requirements set by their leaders. The case of blood donation is a prime example—if leadership places significant emphasis on this activity, employees are likely to follow suit. Smith and Lee (2) have found that employees cite “trust in leadership” as a key motivator. A perceived lack of leader involvement often results in perceived disinterest. Their study demonstrates that proactive, visible leadership, when combined with logistical support and persuasive communication, significantly enhances workplace blood donation rates. Browne et al. (3) confirm that a strong commitment from organizational leaders markedly increases employee participation in blood donation programs, synthesizing empirical evidence to quantify this effect. Moreover, fear and misconceptions about donation diminish when leaders address concerns openly.

In some organizations, blood donation has become a routine activity. Leaders often participate themselves, setting a precedent for their staff. For many employees, seeing their leaders donate blood inspires them to engage in similar behavior. Lee and Ho (4) highlight how social recognition and leadership support can bolster employee participation in workplace blood donation programs. Leadership support is crucial: active endorsement and participation in blood drives by leaders significantly increase employee engagement. The study suggests that HR and management should integrate recognition programs and leadership involvement to sustain blood donation initiatives.

Leaders typically transmit their values and beliefs to employees through subtle yet consistent forms of influence. This gradual, ongoing process helps embed these ideas deeply within the workforce. Schein (5) posits that for founders, leaders, managers, and even some senior employees within organizations, focusing on specific issues is the most effective mechanism for the communication of information. It is crucial for leaders to maintain a sustained focus on a particular issue rather than the intensity of their focus. During meetings or activities dedicated to planning and budgeting, founders and leaders of organizations communicate key concerns to their employees. If the beliefs, values, and assumptions of leaders are consistent, the mechanisms of cultural implantation interact and reinforce each other, a phenomenon clearly reflected in the leaders' daily behaviors.

Dongying (6) emphasizes the necessity of enhancing the attention paid to blood donation by organizational leadership. Gao Dongying stresses the importance of leaders' attention to their responsibilities in group blood donation. She suggests that the quality of work in this area should serve as an indicator for evaluating leadership performance and that a system of accountability for leaders should be established. Blood donation coordinators at various units should receive encouragement to boost their enthusiasm for organizing and promoting the activity.

### 2.2 Competence of blood donation coordinators

Leadership plays a pivotal role in the success of group blood donations. However, the competence of the designated blood donation coordinator is equally essential for effective implementation. This individual oversees the entire process, from recruitment and mobilization to organization and execution, and must possess the necessary skills for these tasks. Qing et al. (7) note that the role of recruiting blood donors has evolved into a specialized profession requiring skills in communication, public relations, and marketing.

An exemplary blood donation coordinator should demonstrate genuine passion and enthusiasm for their work. Additionally, they require strong mobilization and persuasion skills, capable of uniting people and fostering a genuine interest in blood donation. According to Kouzes and Posner (8), effective leaders clarify their values and exemplify these through consistent actions. They build trust by aligning their behavior with shared values, envision an uplifting future, and enlist others by appealing to shared aspirations. Leadership involves teamwork; exceptional leaders promote collaboration and empower others, while recognizing contributions and celebrating achievements to strengthen commitment.

In comparison to organizational leaders, blood donation coordinators interact more directly with employees. Their personal attitude toward the work and the specific methods they employ for donor mobilization significantly impact the effectiveness of donor recruitment. Johnson and White (9) find that direct supervisor support, more than organizational policies or top leadership, is crucial in motivating employees to donate blood. Employees were 2.5 times more likely to donate if their immediate supervisor actively encouraged participation.

### 2.3 The motivational impact of incentives for blood donation

Blood donation is a commendable act with irreplaceable social value. As blood cannot be artificially synthesized and must be sourced from healthy, eligible donors, the contributions of these individuals are crucial for maintaining a stable blood supply for clinical needs. The World Health Organization announced “Blood Donation: Passing on Hope, Saving Lives Together” as the theme for World Blood Donor Day 2025. This theme underscores the direct life-saving impact of blood donation and calls on all sectors of society to participate in ensuring a stable blood supply by enhancing awareness campaigns and optimizing related policies to increase donation rates. Numerous studies have consistently highlighted the role of incentives in encouraging blood donation. It is essential to clarify that incentives do not represent the sole motivation for donors; rather, they signify that the noble act of blood donation merits significant recognition and encouragement. Therefore, targeted preferential policies and benefits should be provided to blood donors as a tangible acknowledgment of their vital contributions to public health.

Dongying (6) observes that the provision of subsidies and rewards has established a conventional work pattern and mindset among entities. To enhance the enthusiasm of Beijing's residents for participating in voluntary blood donation, societal recognition in the form of spiritual rewards is necessary to promote humanitarianism and advocate a new social ethos.

Lacetera et al. (10) discover that extrinsic incentives can coexist with intrinsic motivation, such as altruism, in this context. Incentives increased first-time donor turnout by 26% and retained existing donors more effectively than the absence of rewards. Rewards were particularly effective for less frequent donors.

Incentives provided to donors prior to blood donation, the experience during the donation, and post-donation care are equally important. Incentive policies should form a comprehensive system covering the entire blood donation process. The World Health Organization (11) advocates promoting altruism and community engagement over paid donations to ensure safety and sustainability. Recommendations include improving the donor experience (e.g., reducing wait times, conducting post-donation follow-ups), and implementing loyalty programs (e.g., milestone awards) that do not involve monetary incentives. Governments are encouraged to integrate these donor programs into national health policies with dedicated funding.

According to the International Federation of Red Cross and Red Crescent Societies (12), corporations contribute through employee donations while simultaneously gaining benefits related to Corporate Social Responsibility (CSR). Non-monetary incentives, such as recognition certificates and flexible time-off for donors, enhance altruism without compromising it. Corporations should highlight their impact by stating contributions quantitatively (e.g., “Our company contributed 500 units this year”) and provide post-donation care (e.g., offering snacks and rest areas) to improve the donor experience.

Appropriate and appealing incentives play a crucial role in attracting a larger number of potential donors, which helps expand the donor pool and fosters a sustainable blood donation culture. Titmuss (13) demonstrates that voluntary donation systems enhance social trust and collective responsibility. These systems tend to yield higher-quality blood donations because donors lack a financial motive to hide health risks. Titmuss challenges the neoliberal assumption that markets optimize all resources, arguing that blood is a unique commodity where market incentives can backfire and economic rewards may undermine intrinsic motivation.

### 2.4 The engagement level of blood donors

Altruism is the predominant motivation for blood donation. Donors voluntarily contribute blood to assist patients in need, thereby replenishing a renewable medical resource that saves irreplaceable human lives. Donors often experience a profound sense of accomplishment and pride from their act of helping others. If the donation process is comfortable, this positive behavior is likely to be sustained. Heartfelt motivation tends to be more enduring. Expressions such as “saving lives” or “helping others” are particularly strong predictors of altruism. Moreover, moral obligations or religious and cultural beliefs also serve as personal motivators for donating blood. However, factors such as needle phobia, fear of fainting, or past negative experiences, along with logistical issues like time constraints, poor location access, or long wait times, can significantly decrease attendance rates (14).

Misje et al. (15) analyze the psychosocial factors that influence voluntary blood donation, focusing on sustained donor commitment rather than sporadic contributions. Altruism and social responsibility are primary motivators for active donors. Ferguson et al. (16) suggest that acts of help are more likely to be reciprocated when perceived as genuinely benevolent rather than driven by external pressures or selfish motives. Participants reciprocated more when the helper's actions were framed as autonomous and kind.

Group donors often participate in blood donation during working hours, which necessitates a careful balance between donation activities and regular work responsibilities. Thus, when organizing blood donation drives, organizations must consider the work characteristics and schedules of employees to ensure they are provided with appropriate rest periods post-donation. For jobs that are physically demanding, an extended rest period should be provided. Thoughtful planning can alleviate concerns employees may have about donating blood. Wilson and Ferguson (17) note that employees perceive donating as disruptive to their work schedules, citing concerns such as “lost productivity” and strict break policies. Additionally, inadequate communication about blood donation can lead to reduced participation, as can worries about post-donation fatigue impacting job performance. Charbonneau et al. (18) find that fears of contracting illnesses (e.g., the flu) decrease blood donation rates, while group challenges within a company (e.g., department competitions) can increase donations. Employees show a preference for donating during mid-week lunch hours rather than early mornings or weekends.

Blood donation is also a social behavior that is embedded in interpersonal interactions. Donors are influenced by those around them, with the experiences of family members, colleagues, or friends having a significant impact, especially on first-time donors who tend to trust and follow the advice or recommendations of these close peers. The sense of achievement from saving lives also motivates repeat donations. Occasionally, donors may be temporarily unable to donate due to health reasons, reflecting an objective reality. Gillespie and Hillyer (19) suggest that personal connections (e.g., a family member or friend needing blood) increase the likelihood of donation. However, fear (of needles, pain, or fainting) deters potential donors, as do a lack of awareness about blood needs or eligibility criteria, and inconvenience related to time, location, or a poor donor experience. Self-efficacy, or the belief in one's ability to donate, plays a key role, as do social norms which include peer and family influence. Bednall and Bove (20) point out that some donors view donation as a civic or ethical duty, and that family traditions or community engagement can encourage participation. While motivations drive donations, barriers such as fear, inconvenience, and lack of awareness often prevail.

Bednall and Bove (14) summarize that temporary deferrals due to factors such as low iron levels or recent travel are not conducive to blood donation. Negative past experiences, such as fainting or poor treatment by staff, also decrease the blood donation rate. Donors are more influenced by altruism, social norms, and past positive experiences.

### 2.5 Other environmental factors influencing blood donation

Blood donation is a form of social behavior. It is significantly influenced by the broader social environment. Gordon (21) provides a compelling model for sociological research through situational analysis. By constructing models of typical situations, social scientists can interpret specific events as instances of these typical situations.

Some organizations encounter considerable difficulties when mobilizing staff for blood donation. In some instances, demanding work schedules prevent employees from finding time to donate. Additionally, doubts and concerns about the process of donation can be caused by negative public opinion or misinformation. Dongying (6) identifies, through a survey, that the primary challenges faced by entities in facilitating blood donation include employees' concerns about the act of donating, the incentives provided to donors, and the demanding nature of their work.

It is crucial to maintain a positive image of voluntary blood donation and to sustain donors' sense of identification with and pride in this altruistic act, which are key to reinforcing repeat participation. Qing et al. (7) argue that the tangible aspects such as the donation environment, collection equipment, service facilities, staff, promotional materials, and small mementos are concrete despite the abstract concept promoted by voluntary blood donations. These tangible elements act as physical cues through which donors perceive the intangible concepts associated with blood donation.

According to the literature review, readers can discern the factors from four sections that influence the mobilization of group blood donation. However, previous research has not identified the most critical factor. In the subsequent chapter, a survey will be conducted to test the core influencing factor. This research is expected to yield new findings and results that will elucidate the crucial factors influencing the successful mobilization of group blood donation.

## 3 Research design

Brewer (22) argues that there is a foundational unity in two key aspects despite significant diversity in research content and methodologies across various disciplines within the social sciences. First, these disciplines converge on a shared theme involving the social attributes of culture, markets, and governance. Consequently, the methodologies employed may vary, with each emphasizing different facets of these attributes. Second, these fields are united by common public values that stem from this shared theme. Emphasizing these public values can highlight this fundamental unity.

Currently, the coordination of group blood donation sessions is primarily managed through telephone appointments. As the person overseeing these appointments, the author maintains close communication with coordinators from various organizations, developing a thorough understanding of each group's blood donation patterns. If the number of donors from a particular organization shows a significant deviation from previous years, the author typically inquires about the reasons during the appointment process. Some organizations attribute this change to new leadership that places a stronger emphasis on blood donation initiatives, while others cite enhanced post-donation benefits that have significantly increased volunteer participation. Occasionally, the actual number of blood donors falls short of the scheduled appointments. In such instances, the author consults with the organization's blood donation coordinator to determine the cause, whether it is due to low turnout among registered participants or other factors. Through years of direct interaction with donation coordinators, the author has developed a keen interest in identifying these core factors through a dedicated survey, aiming to promote effective practices that will enable organizations to organize group blood donation activities more successfully.

The process of group blood donation typically unfolds as follows: Initially, an organization receives an official request from the Beijing Blood Donation Office, which seeks coordination for a group blood donation event. In response, the leadership of the organization appoints a staff member to oversee the implementation of this activity. This coordinator is charged with the tasks of mobilizing and recruiting potential donors from within the organization and informing them about the incentives associated with blood donation, such as time off, small gifts, or other benefits. Employees then decide whether to participate, considering their personal circumstances and the information provided. This sequence, which extends from the receipt of the official notice to the actual donation, constitutes the entire workflow of group blood donation. The success of an organization's group blood donation event depends significantly on each step of this process. Despite variations in the types and sizes of these entities, the components that facilitate the organization of blood donations remain consistent. However, a critical question persists: Which step is most pivotal? This study aims to address this question comprehensively. Building on the described workflow, the author has identified four key factors that may influence the success of group blood donations: the level of importance attributed to blood donation by entity leadership, the recruitment capabilities of the coordinator, the incentives provided to donors within the organization, and the participation level of the donors. The study encompasses 100 entities that participated in group blood donation from 2018 to 2024. These entities organized annual group blood donation events, although some suspended their activities for 1–2 years during the COVID-19 pandemic from 2020 to 2022, subsequently resuming their annual activities post-pandemic. A survey was subsequently designed to ask participating organizations to evaluate and rank these four factors, aiming to identify which they perceive as the most critical in influencing the success of group blood donation activities.

Following the assessment of these factors, the researcher investigated the impact of the COVID-19 pandemic on group entities during 2020–2022. The outbreak significantly affected group blood donations, with the degree of impact varying according to local epidemic prevention policies in Beijing, which aimed to minimize mass gatherings. Being a concentrated blood donation activity, group blood donations were particularly affected. Despite these challenges, some entities continued their group blood donation efforts, managing to maintain donation levels despite the pandemic.

## 4 Methods and data

Currently, there are over 1,000 registered blood donation organizations in Beijing specializing in group donations. With a decade of experience in coordinating and managing these donations, the author has gained significant firsthand knowledge of the operational practices of various institutions involved. To ensure the accuracy and credibility of this study, the author randomly selected 100 organizations that have consistently organized group blood donations from 2018 to 2024. These organizations demonstrate long-term engagement and have developed mature models for planning and executing their activities. In contrast, some organizations participate in group blood donations only sporadically. Their irregular involvement fails to provide the consistency necessary to develop substantial organizational knowledge. Consequently, these groups cannot offer reliable insights into the primary factors influencing group blood donation. The selected 100 organizations, however, exhibit a high degree of stability and consistency. Their feedback during the research process offers insights that have been tested and validated over time, providing valuable guidance and replicable best practices for other blood donation groups.

Group blood donation appointments are coordinated via telephone, providing the author with ample and flexible opportunities for direct communication with the coordinators from each organization. The survey was conducted between late 2024 and early 2025. It involved interviewing some coordinators during the scheduling of their group donation appointments and others after the completion of their events. All interviews were conducted in a relaxed, informal setting to foster open dialogue. The survey focused exclusively on four key influencing factors and did not collect personal or sensitive information about the coordinators. This approach contributed to an exceptionally high willingness to participate, as no respondents declined to answer. As the coordinators are directly responsible for organizing and managing their organizations' blood donation activities, they possess a deep understanding and practical insights into the four examined factors. Additionally, motivated by a shared objective to enhance group blood donation efforts, respondents were candid and proactive in sharing their experiences and perspectives. The author clearly communicated to all participants that the research was conducted for academic purposes only, aiming to summarize and promote effective practices in group blood donation. To further ensure trust, all data and results related to the participating organizations were treated with strict confidentiality.

Data were gathered through telephone interviews, during which the leadership's prioritization of blood donation efforts, the mobilization and organizational capabilities of the blood donation coordinators, the feedback provided to blood donors within the entity, and the engagement levels of the blood donors were assessed. Entities were requested to rank these four factors in order of importance, with the most critical factor ranked first and so on. The evaluation criteria included the following elements: Leadership's Emphasis on Blood Donation: This encompasses the entity's leadership's attention to and support for blood donation activities, including the provision of time and space priorities and conveniences for the activities, as well as both material and moral incentives offered to blood donors. Mobilization and Organizational Capabilities of the Blood Donation Coordinators: This involves employing various promotional and recruitment strategies, clearly explaining the significance, knowledge, and procedures of blood donation, and timely compilation and organization of registration numbers for blood donation events. Feedback Provided to Blood Donors within the Entity: This includes feedback that blood donors deem acceptable or necessary, such as granting days off post-donation, providing nutritional supplements, or incorporating these contributions as criteria or reference factors in employee evaluations and recognitions. The specifics of the feedback are determined independently by each blood donation entity based on their circumstances. Engagement Levels of Blood Donors: This refers to whether blood donors are well-informed about blood donation, whether they harbor any misconceptions or are influenced by negative information which might lead to a negative attitude toward blood donation, and whether they have the time and physical ability to donate blood. The results of the survey are presented in Table 1.

**Table 1:** Ranking of importance of blood donation impact indicators by 100 group blood donation entities.

**Impact Indicator**	**Ranked 1st**	**Ranked 2nd**	**Ranked 3rd**	**Ranked 4th**	**Total**
Leadership's prioritization of blood donation	79	15	4	2	100
Feedback provided to blood donors	12	33	22	33	100
Engagement levels of blood donors	7	26	52	15	100
Mobilization and organizational capabilities of the blood donation coordinators	2	26	22	50	100

## 5 Results analysis

In the study of 100 entities, participants were asked to rank four key factors concerning their entity's involvement in blood donation: the leadership's prioritization of blood donation, the mobilization and organizational capabilities of the blood donation coordinator, the feedback provided to blood donation participants within the entity, and the engagement levels of blood donors. The findings reveal that 79 entities ranked the prioritization by leadership as the most critical factor influencing their blood donation efforts. This underscores the pivotal role of leadership commitment in the promotion and sustained attention to the blood donation process; where leadership is engaged, the initiative is more likely to be effectively implemented and supported across various departments or branches. Conversely, a lack of leadership commitment could potentially preclude the initiation of blood donation activities, highlighting the central influence of leadership prioritization.

Additionally, 33 entities selected the feedback and incentives provided to blood donors as the second most important factor. These entities offer appropriate compensation and care to their blood-donating employees, fostering a sense of support and motivation. The encouragement provided by these entities is attractive and valued by employees, serving both to assist patients in need and to benefit the donors themselves. It was observed that participation in blood donation could result in several days off, although special rest is not typically necessary after donating blood. The provision of leave and material incentives by the entity not only acknowledges but also encourages the donors' contributions.

Conversely, another 33 entities ranked the incentive feedback as the least important of the four factors, indicating a significant divergence in perspectives. This contrast presents a fascinating phenomenon within the dynamics of group blood donation, suggesting that the role of material incentives remains a topic worthy of further exploration.

Fifty-two entities ranked the engagement levels of blood donors as the third most important factor. While acknowledging the significance of donor participation, these entities consider it less critical compared to the two predominant influencing factors that precede it. Group blood donation, often construed as an organizational behavior, sometimes parallels an administrative action or requirement within an entity. Individuals within an organization are expected to comply with management and support the entity's blood donation initiatives. Although the engagement levels of blood donors in group blood donations are crucial, they are deemed less significant than those of individual donors, due to the organizational nature of group donations, where employees are primarily expected to adhere to the directives of the entity.

Ranked last among key influencing factors is the recruitment and mobilization capability of the blood donation coordinator, selected by 50 entities as the fourth most significant factor. Recruitment and mobilization strategies akin to marketing are more effective for individual blood donors, as some individuals excel in persuading and mobilizing others, often due to personal charisma or a compelling appeal. Such traits make these individuals particularly suitable for recruiting blood donors. However, as discussed earlier, group blood donation is fundamentally an organizational behavior reliant on administrative support. Consequently, the scope for a blood donation coordinator to influence outcomes is limited. Effective mobilization still heavily depends on leadership support and the strength of incentives offered for blood donation. Without these elements, the recruitment capabilities of the blood donation coordinator are considerably constrained.

An in-depth analysis of 79 organizations revealed that leadership emphasis on blood donation is perceived as the most critical factor influencing its success. Among these organizations, 30 identified incentives or rewards for donors as the second most important factor, while 28 ranked it fourth. This distribution is consistent with the pattern observed in Table 1, which encompasses all 100 surveyed organizations, suggesting that strong leadership tends to diminish the relative importance of incentives and rewards. Effective leadership can secure more resources, thereby enhancing the provision of donor rewards and increasing employee participation. Even without additional incentives, group blood donation, being a highly organized collective activity, generally instills a sense of obligation among employees to adhere to leadership directives. Further analysis of 15 organizations that ranked leadership emphasis as the second most important factor offers additional insights. Ten of these organizations considered donor incentives as the most crucial factor, underscoring that the provision of such incentives is ultimately determined by leadership. Three organizations identified donor willingness as the primary factor; however, this becomes less significant under strong leadership directives. The remaining two organizations focused on the competence of the donation coordinator, who is appointed by the leadership. Collectively, these findings underscore the pivotal role of leadership in influencing the outcomes of group blood donation initiatives.

In addition to these primary factors, several other elements can affect group blood donation activities. Environmental factors, such as the timing of major events, frequently necessitate the rescheduling or postponement of planned blood donation sessions. Short-term disruptions, including natural disasters like floods or blizzards, also impact these activities; however, these interruptions are typically temporary, and normal operations usually resume once the adverse conditions abate. Data analyzed from 100 entities during the 2020–2022 pandemic period indicate that 5% of these entities were affected each year, either by failing to organize blood donation events or by experiencing significantly lower-than-expected donor turnout. The remaining 45% of organizations were affected for varying durations, with some impacted for 1 year and others for 2 years.

Additionally, the researcher observed that during the 3 years of the pandemic (2020–2022), 50% of the entities were not affected by the pandemic and successfully organized and completed their blood donation quotas. Some entities managed to complete their annual blood donation tasks promptly during periods when the pandemic's impact was less severe, or by organizing smaller, more frequent donation sessions throughout the year. It was also noted in the study that entities that prioritized leadership attention to blood donation activities were among those that managed to overcome adverse conditions effectively. This underscores the crucial role of entity leadership in prioritizing blood donation; a committed leadership can significantly influence the continuation and success of these activities even under challenging circumstances.

## 6 Conclusion and discussion

The recruitment, mobilization, and organizational implementation of group blood donation initiatives are complex undertakings that transcend the simplistic notion of merely gathering individuals to donate blood. The successful execution of a blood donation event is contingent upon a multitude of factors. Predominantly, it involves core factors and environmental factors that collectively influence the process of group blood donation.

Within the realm of core factors pertinent to group blood donation, a retrospective study was conducted focusing on several critical aspects: the leadership's prioritization of blood donation, the competencies of the blood donation coordinators, the feedback provided to donors by the entity, and the engagement levels of blood donors to participate in the donation process. This study encompassed 100 entities that participated in blood donation from 2018 to 2024. Over these seven years, amidst various societal changes, it was observed that 79% of these entities ranked the prioritization of blood donation by their leaders as the most significant factor. Entities where the completion of blood donation was unaffected or minimally impacted consistently demonstrated significant leadership attention and concern toward blood donation. The degree of importance placed on blood donation by entity leaders emerged as the most crucial factor in the resilience of group blood donation efforts.

The leadership of organizational entities plays a pivotal role in influencing financial control and personnel arrangements. This authority extends to enhancing the incentives provided to blood donors, thereby attracting a greater number of participants. The attention given by organizational leaders significantly boosts the engagement levels of blood donors. Consequently, maintaining effective communication with these leaders is crucial during group blood donation initiatives. By mobilizing and organizing blood donation activities at the leadership level, employees perceive these actions as integral to the organization's responsibilities, prompting voluntary participation.

The capabilities of a blood donation coordinator, while crucial, are considered less influential in the initial stages of donor recruitment and mobilization for group blood donations, which differ fundamentally from individual donations. In the case of individual donations, the varied abilities of staff can lead to diverse recruitment outcomes. However, group blood donations represent a structured activity predominantly led by organizational leaders. The skills of a blood donation coordinator are primarily manifested at the operational phase, such as scheduling donation times for donors, managing onsite processes and order, and overseeing post-donation registration and care. Thus, a proficient blood donation coordinator is vital for the successful execution of the donation event, but their importance is more pronounced during the operational stages rather than in the critical phases of donor recruitment and mobilization.

The second most crucial factor is the feedback provided to blood donors. Blood donation is a voluntary act, and when the methods and magnitude of incentives create an appealing proposition, they significantly enhance recruitment outcomes. Hence, feedback to blood donors is paramount. According to a survey, 33 entities acknowledge the importance of tangible rewards, unequivocally affirming their effectiveness. Conversely, another 33 entities deem material incentives as less critical, highlighting the significance of administrative directives in group blood donation contexts. This disparity underscores that the presence of material incentives may not be as crucial in the context of administratively driven group blood donation efforts.

In the context of group blood donation, the social environment plays a pivotal role as an environmental factor. A thriving, positive public opinion within the social environment significantly enhances the effectiveness of group blood donations. Conversely, environments saturated with negative discourse surrounding blood donation can severely damage the outcomes of such group initiatives. Furthermore, the global pandemic in recent years has exerted a considerable impact on group blood donations, although some entities have successfully conducted blood donation drives, largely attributable to the prioritization given by their leadership.

Previous studies on group blood donation have typically focused on one or two influencing factors in isolation, such as the impact of leadership emphasis, the extent of incentives provided to donors, or the donors' own awareness and acceptance of blood donation. However, these studies lacked a systematic approach to identifying and ranking the full spectrum of factors throughout the entire group blood donation process. By contrast, this study adopts a workflow-oriented perspective (grounded in the complete organizational process of group blood donation outlined earlier), enabling the accurate identification of all relevant influencing factors and a horizontal comparison to determine the most critical ones. Beyond internal organizational factors, this study also incorporates a key external variable: the broader social environment. Notably, all participating organizations experienced the 3-year COVID-19 pandemic—a context of extreme external challenges. Within this framework, the study explores which internal factors, when absent, led to severe disruptions in group blood donation, and which internal factors ensured resilience, allowing donations to continue unaffected. The findings reveal that both the core internal factors influencing group blood donation and those enabling resistance to adverse external conditions ultimately stem from the organizational leadership's emphasis on blood donation. This conclusion reflects the comprehensive and systematic insights derived from this study.

For more positive developments in group blood donation, it is imperative to bolster efforts in mobilizing, communicating with, and supporting the leadership of blood donation entities. Should these entities face practical challenges, the initial step involves a detailed analysis with the principal leaders of the difficulties and characteristics of organizing blood donation drives, thereby facilitating a smooth execution of these activities within the entity. In the broader societal context, efforts should be made to foster a conducive social atmosphere for group blood donations and to minimize the emergence of adverse public opinions related to blood donation. From the perspective of the entity, it is essential to recognize and encourage employees who participate in blood donation. According to the findings presented in this study, the primary determinant for the successful implementation of group blood donations is the degree of emphasis placed on the initiative by the entity's leadership.

## Data Availability

The original contributions presented in the study are included in the article/supplementary material.
